# Complementary and Integrative Health Approaches for Weight Management in the Obese Population: The 2018 Korea National Health and Nutrition Examination Survey

**DOI:** 10.3390/ijerph18158161

**Published:** 2021-08-02

**Authors:** Sang-Dol Kim

**Affiliations:** Department of Nursing, College of Health Science, Kangwon National University, 346 Hwangjo-gil, Dogye-eup, Samcheok-si 25949, Kangwon-do, Korea; nu11110@kangwon.ac.kr

**Keywords:** obesity, complementary and integrative health, national health and nutritional examination survey, weight management

## Abstract

(1) Background: Obesity management has become an important issue due to the COVID-19 outbreak; therefore, periodic surveys on the approaches to obesity management of the entire population and target obese population are required. (2) Methods: The study used nationally representative data from the 2018 Korea National Health and Nutrition Examination Survey. Participants reported all approaches they had used to reduce or maintain weight in the past year. Data were analyzed with multiple response methods. (3) Results: The most commonly reported approach was exercise, which included fitness, yoga, biking, and other physical activities (74.7% of respondents), and the second most commonly reported approach was decreased food intake (69.6% of respondents). The use of approaches differed according to respondents’ demographic characteristics. Regarding sex-related differences, in particular, men preferred to exercise, while women were more likely to decrease food intake. Among men, exercise was highest in the 40–49 years age group (48.3%). Among women, decreased food intake was highest in the same age group (16.1%). (4) Conclusions: These findings indicate that it is necessary to introduce individualized weight management approaches and measures according to target groups in obese adults.

## 1. Introduction

The prevalence of obesity has reached epidemic proportions worldwide [1]. The World Health Organization (WHO) has pointed out the various problems caused by the continued increase in the global prevalence of obesity [2]. In many parts of the world, obesity is a more serious health issue than malnutrition [1,2]. According to previous studies, obesity is a risk factor for many acute and chronic health conditions, including type 2 diabetes, heart disease, and cancer [1,2,3]. Furthermore, it has been reported that obesity is associated with immune vulnerability [4]. A recent meta-analysis study suggested that obese individuals tend to have a higher mortality rate from the COVID-19 pandemic than those who do not [5]. According to the WHO, “at least 2.8 million people die each year worldwide as a result of being overweight or obese, and an estimated 35.8 million of global disability-adjusted life years are caused by overweightness or obesity” [2]. Obesity is thus considered a metabolic disease and “a gateway to not only ill health but also elevation of morbidity, mortality, and chronic complications” [2,6,7,8]. In addition to the medical burdens, obesity has led to financial burdens worldwide [2,9,10,11]. One previous study has estimated that “obesity-related medical costs will increase by $48–66 billion/year in the USA and by £1.9–2 billion/year in the UK by 2030” [9]. In South Korea, socioeconomic losses due to obesity amounted to USD 10.3 billion in 2016, which is a 1.6-fold increase over the past three years [10].

According to the data mentioned above, the global prevalence of obesity continues to increase, with a worsening trend in health and other associated problems. Regarding this situation, a prior study suggested that new innovative approaches for obesity prevention be introduced [12]. Another previous study pointed out that the government’s role in obesity management was insufficient concerning interventions and policies to provide evidence-based obesity care and emphasized the need for continuous national surveillance for the same [13,14]. It estimated that the promotion and dissemination of weight management strategy programs for people who are obese and those vulnerable to it should be strengthened. Moreover, it expressed the need for continuous and rigorous national surveillance for evidence-based obesity management.

In general, the cause of obesity is considered to be an adherence to unhealthy diet and lifestyle patterns [15]. In addition to medicines, obesity control strategies depend primarily on a healthy lifestyle that incorporates adequate physical activity and food intake to maintain energy balance [16]. In recent years, access to complementary and alternative medicines for obesity management has increased significantly [15]. Many obese individuals may prefer and actively adopt complementary or alternative health approaches and nonpharmacological treatment options for weight management, despite remarkable weight control-related advances in conventional medicine [1,3,6,17]. According to a 2012 national survey in the United States, more than 30% of American adults used complementary and integrative health (CIH) approaches including, “natural products, mind and body practices, and other methods to manage their weight” [17]. A 2014 survey in Jordan found that approximately 40% of women aged 18 years or older used some form of complementary and alternative medicine for weight control [6]. Australian researchers have reported that three-quarters of a group of breast cancer survivors had used complementary therapies for weight loss [18]. Excluding these previous studies, few have published national survey data on CIH approaches used in weight control.

Meanwhile, obesity management has recently been highlighted as an important public health issue due to the COVID-19 pandemic [5], and therefore, periodic investigations of weight management approaches are required at the national and community level [13,14]. A survey of the CIH approaches for weight management adopted by obese populations is considered to be meaningful in several dimensions. First, this would make it possible to grasp the current status of CIH approaches for weight management. Second, these data enable overall monitoring of CIH approaches for weight management in the future. Third, these findings could be used as basic data to compare weight management before and after the COVID-19 pandemic. In this context, the purpose of this study was to describe the use of CIH approaches for weight management in Korean adults with obesity.

## 2. Materials and Methods

### 2.1. Study Design and Participants

The study was a secondary analysis of data obtained from the 2018 Korea National Health and Nutrition Examination Survey (KNHANES) φ-3 by the Korea Disease Control and Prevention Agency (KDCA) [19]. The methodology used by the KNHANES to obtain a nationally representative sample has been documented elsewhere [19]. Responses were obtained through self-reported questionnaires. Data for this study were downloaded from the KNHANES website (https://knhanes.cdc.go.kr/knhanes/eng/index.do (accessed on 30 March 2020)). A total of 6170 respondents were included in the KNHANESϕ-3 database. For the current analysis, the sample was limited to those aged 19 years and older. After excluding pregnant women, the study sample size was 2142 (1123 men, 1019 women).

### 2.2. BMI Assessment

The body mass index (BMI) was calculated as the ratio of weight and squared height and categorized as underweight (BMI < 18.5), normal weight (18.5 ≤ BMI < 23), pre-obese stage (23 ≤ BMI < 25), obesity class I (25 ≤ BMI < 30), obesity class II (30 ≤ BMI < 35), and obesity class III (35 ≤ BMI) [20]. Height and weight were measured in the KNHANES using digital stadiometers (GL-6000-20; G-tech, Seoul, Korea) and electronic scales (Seca 225; Seca, GmbH & Co, Hamburg, Germany), respectively.

### 2.3. Survey of Weight Management Approaches

The survey was conducted in a mobile screening vehicle. After participants had confirmed that they were the subject of the investigation and had filled out a consent form, the investigation proceeded. The survey was conducted by a professional research team consisting of nurses, nutritionists, and health science majors. After the team had been selected, they completed practical training (theory education and practice) for 2 to 4 weeks and were then put at the survey site. Afterward, the ability to conduct the surveys was continuously verified through on-site quality management, as well as regular training seven times a year.

The weight management survey was completed through self-reported questionnaires. The weight management question asked participants to select all the methods (multiple responses) that they had used to lose or maintain weight in the past 12 months. Response items included the following: exercises (including fitness, yoga, biking, and other physical activities), fasting (going without a meal for more than 24 h), reducing portion sizes or avoiding specific foods, skipping meals, taking prescription medications or nonprescription weight loss products, including herbal medicines, taking health functional food, and following a single-food diet (eating only one food such as grapes, milk, or sweet potatoes) [20].

### 2.4. Data Analysis

Sampling weights to ensure the representativeness of the sample relative to the Korean population were available in the KNHANES database. Missing data were processed as valid values. Frequencies of sociodemographic variables and obesity levels were calculated for sex and age groups. Among subjects over 19 years of age, pregnant women and those taking prescription or nonprescription medications for weight loss were excluded from the data analysis. All analyses were performed using SPSS version 23.0 (IBM Corp., Armonk, NY, USA).

### 2.5. Ethical Considerations

The KNHANES Φ-3 was approved by the Institutional Review Board of the KDCA (2018-01-03-P-A). The raw data used in this study were provided by legitimate procedures through the KNHANES website under the KDCA (https://knhanes.cdc.go.kr/knhanes/sub03/sub03_02_02.do (accessed on 30 March 2020)) [19]. Personal information in the KNHANES data is fully anonymized. The KDCA provides only de-identified data to protect the identity of the participating individuals. This study did not involve any personal identity-based information of the participants. Therefore, the informed consent of the participants was not required for this study.

## 3. Results

The results of the study variables are presented in Table 1, Table 2, Table 3, Table 4 and Table 5. The overall age-adjusted prevalence of obesity (BMI ≥ 25 kg/m^2^) was 35.0%; this was 41.9% among men and 28.2% among women. The prevalence of obesity was highest for men aged 40–49 years (23.0%), and for women aged 70 years and older (20.8%). Among the demographic characteristics, the prevalence of obesity was higher in married individuals than in singles, and in the economically active population group than the economically inactive group. In addition, obesity was more prevalent in men with higher family income and women with lower family income. Considering the level of education, the prevalence was highest in the group with a college degree or higher for men, and the group below elementary school graduation for women.

Among obese respondents, the most commonly used approaches for weight control were exercise (mentioned by 74.7% of respondents), decreased amount of food intake (69.6%), skipping meals (12.7%), health functional food (10.2%), one-food diets (2.6%), herbal medicines (2.5%), and fasting (2.2%). Exercise was the most common approach reported by men (42.7%), while decreased food intake was the most common approach for women (36.2%). Among men, exercise was most common in the 40–49 years age group (18.3%). Among the groups of men, the CIH access rate was in the order of exercise (42.7%), decreased food intake (33.3%), skipping meals (6.2%), health functional food (3.4%), fasting (1.0%), one-food diet (1.0%), and herbal medicines (0.6%). Among women, decreased volume of food intake was most common in the 40–49 years age group (16.1%). Among groups of Korean obese women, the CIH access rate was in the order of decreased food intake (69.6%), exercise (32.0%), health functional food (6.8%), skipping meals (96.5%), herbal medicines (1.9%), one-food diet (1.6%), and fasting (1.2%).

## 4. Discussion

The prevalence of obesity found in this study was 35%, which is higher than the 26.1% measured in 1998 when the KNHANES was launched [21]. This is similar to widely documented increases noted worldwide over this period [1,2,9]. These observations support warnings that global obesity rates are at epidemic levels, and Korean rates clearly indicate a national health problem [22]. In this study, the prevalence of obesity was identified as meaningful results based on demographic variables such as sex, age, marital status, economic activity status, family income level, and education level. This study found a higher prevalence of obesity among men than among women, similar to reports from the WHO [2]. In contrast, surveys have revealed that obesity is generally higher among women in the United States [23]. Interestingly, in this study, the obesity prevalence was highest in 40- to 49-year-old men, following which it tended to gradually decrease. However, the obesity prevalence was highest among women over 70 years of age and showed a tendency to increase with age. The obesity prevalence among Korean adults was found to be higher among those who were married and economically active than those who were not. This suggests that additional research is needed to determine the causative association between weight gain and marriage among Korean men. The obesity prevalence tends to be higher for men with higher family income levels and women with lower income levels. This is believed to stem from the culture that Korean women may be more conscious regarding esthetics than men. This also reflects the need to investigate the effect of the gender income gap on the prevalence of obesity. In addition, there was a difference in the prevalence of obesity between men and women based on the level of education. It was most prevalent in men with a college degree or higher and in women after graduation from primary school. These results emphasize that effective measures to combat obesity must be tailored to fit the needs and preferences of different demographic groups. Furthermore, these findings reflect the need for in-depth additional studies to investigate the differences in obesity prevalence based on demographic characteristics in Korean adults. Nevertheless, these results are expected to be used as basic data for evidence-based obesity management.

Key risk factors for the development of obesity have been reported to include “eating disorders, high energy-dense diets, low physical activity, and adoption of a sedentary lifestyle” [1,16]. The WHO recently recommended the development of preventive and control measures aimed at both the individual and national levels [2]. In this study, exercise (including fitness, yoga, biking, and other physical activities) was the most common method of weight control, particularly among 40- to 49-year-old men, who had the highest prevalence of obesity. Similar results have been reported for middle-aged European men [1]. These observations support the proposed WHO guidelines, which promote “regular engagement in physical activity as a fundamental method for weight control in obese individuals” [2]. International reports, however, have been inconsistent in terms of the most commonly used approaches. Yoga was the most commonly reported complementary and alternative approach for weight control in the United States in 2002 [24], which changed to fish oil and other natural products according to the 2012 National Health Interview Survey [3]. Herbal ingredients in plant food supplements are often used for weight control in European countries [25].

Interestingly, in this study, weight-control approaches showed differences by demographic variables such as sex, age, marital status, economic activity status, family income level, and education level. Among the groups of men, the CIH access rate was in the order of exercise, decreased food intake, skipping meals, healthy functional food, fasting or one-food diet, and herbal medicines. The CIH access rate was the highest in 40- to 49-year-old men with the highest prevalence of obesity. These results can be interpreted positively in terms of obesity management. Surprisingly, the CIH access rate was higher in the married and economically active groups of men—the higher the family income and education level were, the higher was the CIH access rate. It is evident that additional studies are needed to confirm the veritable efficacy of these approaches for obesity management.

Unlike obese Korean men who showed the highest preference for exercise, the CIH access rate in obese Korean women was in the order of decreased food intake, exercise, healthy functional food, skipping meals, herbal medicines, one-food diet, and fasting. The current findings differ from reports on Jordanian women with obesity, who had preferences for commercial dietary supplements, herbal products including green tea, and folk remedies [6]. In another previous study, the most popular complementary therapies for weight loss were “dietary supplements (vitamins, minerals, herbs, and antioxidants), yoga, relaxation techniques, massage, and meditation” [18]. The reasons for differing preferences between countries for weight-control approaches likely include cultural eating patterns and irregular daily life habits [26]. There was an age-related difference in the preference for the CIH approach among obese Korean women. The access rate for decreased food intake was the highest, observed in the group of women aged 40 to 49. The group over 70 years of age, with the highest prevalence of obesity, showed a high reduction in food intake volume, a high preference for exercise, and no fasting at all. In particular, among 30- to 39-year-old women, skipping meals and herbal medicines were the preferred choice of obesity management, compared to other age groups. Exercise was also the most preferred approach for obesity management among 50 to 59-year-old women, compared to other age groups. Married women showed a higher rate of access to CIH than unmarried women, and there was no significant difference in access depending on whether they were economically active. Interestingly, in the group of women with the highest family income, the decrease in food intake volume was the least, while the herbal medicine approach rate was the highest. This may be attributed to the high-cost herbal medicine in Korea being highly preferred as a popular diet approach for obese women who are economically wealthy. Among women who graduated from college or higher, the preference for various CIH approaches except exercise was lower than that of other educational groups. This is consistent with the highest prevalence of obesity among women who graduated from primary school or younger. These data can be used as the basis for developing interventions for evidence-based obesity management. From the results of this study, it was confirmed that the preference of the CIH approach for obesity management in the Korean obese population differs based on demographic characteristics. However, research to confirm the degree of effectiveness of the CIH approaches used by the Korean obese population for obesity management should be continued. These results may aid in the reestablishment of the government’s obesity management policy and initiation of its dissemination along with the development of evidence-based interventions.

Many of the practices noted in this study can lead to other health problems. For example, younger women are particularly prone to following unhealthy practices such as skipping meals [27]. Among older individuals who are taking multiple medications, the use of herbal products may be contraindicated [6]. Severe dieting practices can also reduce the cognitive ability to “distinguish hunger and satiety cues, leading to binge eating” [28]. The predominant CIH approaches for weight control by Koreans with obesity are exercise and diet, which reflects global trends [29,30,31,32,33]. A meta-analysis of weight loss studies stressed the role played by the choice of dietary intervention in weight loss [34]. The WHO has emphasized the need to create atmospheres in which individuals with obesity have easy access to healthy food and regular physical activity [2]. The National Center for Complementary and Integrative Health guidelines also recommend a variety of dietary supplements for weight control [35].

Surprisingly, only a few published studies have reported survey data on weight-control approaches in the obese population at the national level. For this reason, the present study has limitations, in that it is difficult to compare the weight management methods of the obese population in Korea with CIH approaches for weight management of the obese population applied in other countries. Another limitation of this study is the structure of questions in the KNHANES. In particular, the response of “exercise” included fitness, yoga, biking, and other physical activities. Future investigations should measure this in more detail by subdividing the “exercise” item into individual items.

Despite these limitations, it should be noted that this study was based on data obtained from a rigorous national health survey. In addition, this information, particularly about differences according to demographic variables such as sex, age, marital status, economic activity status, family income level, and education level, could be useful in assessing the efficacy of specific approaches. The results could be used in follow-up studies to confirm the progress of weight management in these study participants. Finally, the KNHANES data are sufficiently rigorous to enable comparisons across countries and to be used for health policy development. If the sharing of data on obesity management accumulated in various countries is activated, this could aid the derivation, application, feedback, and improvement of global obesity management policies. CIH approaches for weight control must be continuously developed with consideration of “social, cultural, behavioral, and environmental contexts” in the future [36]. Furthermore, evidence-based CIH approaches for individuals with obesity should be continuously tested using rigorous randomized controlled trials [1,13,36]. Above all, based on reliable and representative national survey data and evidence-based strategies, measures for individuals with obesity should be developed and shared, such that they can be implemented in different countries and population groups globally.

## 5. Conclusions

In conclusion, this study analyzed nationally representative data on Korean use of CIH approaches for weight control. Overall, exercise (including fitness, yoga, biking, and other physical activities) was the most common approach used for weight management. The use of the approach differed according to demographic variables such as sex, age, marital status, economic activity status, family income level, and education level. In particular, regarding sex-related differences, men preferred to exercise, while women were more likely to decrease food intake. These findings indicate that it is necessary to individualize weight management approaches and measures according to the targeted demographic characteristics in adults with obesity. Further investigations are needed to verify the efficacy of CIH approaches used by the Korean obese population. Furthermore, these findings could be used as basic data to compare weight management before and after the COVID-19 pandemic.

## Figures and Tables

**Table 1:** Distribution of weight according to BMI in Korean population (≥19 years old).

BMI (kg/m^2^)- Categories	Weight-Division	Men		Women		Total	
		*N*	% (SE)	*N*	% (SE)	*N*	% (SE)
BMI < 18.5	Low weight	51	2.1 (0.3)	164	5.3 (0.5)	215	3.7 (0.3)
18.5 ≤ BMI < 23	Normal weight	849	30.7 (1.0)	155	47.4 (1.0)	2400	39.0 (0.7)
23 ≤ BMI < 25	Pre-obese stage	696	25.3 (1.0)	717	19.3 (0.7)	1413	22.3 (0.6)
25 ≤ BMI < 30	Obesity class I	962	35.2 (1.2)	839	23.1 (0.9)	1801	29.1 (0.8)
30 ≤ BMI < 35	Obesity class II	141	5.8 (0.3)	155	4.4 (0.4)	296	5.1 (0.4)
35 ≤ BMI	Obesity class III	20	0.9 (0.3)	25	0.7 (0.2)	45	0.8 (0.1)
Total		2719	100.0 (0.0)	3451	100.0 (0.0)	6170	100.0 (0.0)

BMI, body mass index; N, number; SE, standard error.

**Table 2:** Demographic characteristics in Korean obese (25≤ BMI) population (age ≥19 years old).

Variables	Men		Women		Total	
	*N*	% (SE)	*N*	% (SE)	*N*	% (SE)
Age group						
19–29	123	16.4 (1.6)	61	9.8 (1.3)	184	13.7 (1.2)
30–39	213	22.3 (1.7)	107	13.7 (1.6)	320	18.8 (1.3)
40–49	235	23.0 (1.5)	172	17.8 (1.5)	407	20.9 (1.2)
50–59	211	19.5 (1.4)	190	20.6 (1.6)	401	19.9 (1.1)
60–69	196	12.0 (1.0)	224	17.4 (1.3)	420	14.2 (0.8)
≥70	145	6.7 (0.8)	265	20.8 (1.5)	410	12.4 (0.9)
Total	1123	59.7 (1.2)	1019	40.3 (1.2)	2142	100 (0.0)
Marital status						
Married	904	44.4 (1.5)	927	35.3 (1.2)	1831	79.4 (1.4)
Single	219	15.5 (1.2)	92	4.8 (0.6)	311	20.3 (1.4)
Total	1123	60.0 (1.2)	1019	40.3 (1.2)	2142	100.0 (0.0)
Economic activity						
Yes	844	48.7 (1.4)	482	20.2 (1.1)	1326	68.9 (1.3)
No	234	11.2 (0.9)	494	19.9 (1.0)	728	31.1 (1.3)
Total	1078	59.9 (1.3)	976	40.1 (1.3)	2054	100.0 (0.0)
Family income level						
Lower	135	5.7 (0.6)	297	10.2 (0.9)	432	15.9 (1.2)
Mid-lower	260	13.6 (1.0)	285	11.8 (0.8)	545	25.4 (1.4)
Mid-upper	349	20.5 (1.2)	241	10.1 (0.9)	590	30.6 (1.5)
Upper	376	20.3 (1.2)	192	7.9 (0.7)	568	28.1 (1.5)
Total	1120	64.0 (1.2)	1015	40.0 (1.2)	2135	100.0 (0.0)
Education level						
≤Primary school	113	4.1 (0.5)	336	11.5 (0.9)	449	15.5 (1.1)
Middle school	95	3.8 (0.5)	138	5.0 (0.5)	233	8.8 (0.8)
High school	386	22.8 (1.2)	281	13.1 (0.8)	667	35.8 (1.4)
College≤	484	29.2 (1.5)	221	19.6 (0.7)	705	39.8 (1.6)
Total	1078	59.9 (1.3)	976	40.1 (1.3)	2054	100.0 (0.0)

N, number; SE, standard error.

**Table 3:** CIHA for weight management according to general characteristics in the Korean obese population (≥19 years old).

Variables	Exercise	Fasting	Decreased Food Intake	Skipping Meals	Herbal Medicines	Health Functional Food	One-Food Diet	Total
Sex								
Men	42.7	1.0	33.3	6.2	0.6	3.4	1.0	52.3
Women	32.0	1.2	36.2	6.5	1.9	6.8	1.6	47.7
Total	74.7	2.2	69.6	12.7	2.5	10.2	2.6	100.0
Age group								
19–29	7.5	0.1	7.9	1.8	0.3	1.3	0.5	9.1
30–39	11.9	0.9	11.7	2.9	0.9	2.1	0.3	15.7
40–49	15.6	0.6	15.2	3.6	0.5	2.5	0.5	21.1
50–59	14.8	0.2	12.4	2.2	0.5	1.8	0.5	20.0
60–69	14.3	0.4	12.0	1.3	0.2	2.0	0.8	19.5
≥70	10.9	0.1	10.5	0.9	0.1	0.5	0.1	14.6
Total	74.7	2.2	69.6	12.7	2.5	10.2	2.6	100.0
Marital status								
Married	63.0	1.9	58.1	10.1	2.1	8.6	2.2	85.5
Single	11.8	0.3	11.5	2.6	0.4	1.6	0.4	14.5
Total	74.8	2.2	69.6	12.7	2.5	10.2	2.6	100.0
Economic activity status								
Yes	48.8	1.8	44.5	9.0	1.7	6.8	2.0	65.7
No	26.0	0.4	25.0	3.9	0.8	3.4	0.6	34.3
Total	74.8	2.2	69.5	12.9	2.5	10.3	2.6	100.0
Family income level								
Lower	11.7	0.3	11.7	2.5	0.1	1.1	0.3	17.0
Mid-lower	18.8	0.5	1.6	2.7	0.4	3.1	0.9	25.1
Mid-upper	22.2	0.8	20.7	3.7	1.0	3.3	0.8	29.7
Upper	20.0	0.6	19.7	4.0	1.0	2.7	0.8	28.2
Total	74.8	2.2	69.7	12.8	2.5	10.2	2.7	100.0
Education level								
≤Primary S.	10.7	0.1	11.6	1.7	0.3	0,7	0.4	16.5
Middle S.	8.5	0.1	7.1	0.7	0.1	0.9	0.3	11.5
High S.	26.6	0.7	24.5	4.5	0.	4.5	1.3	35.4
College≤	29.0	1.4	26.4	6.0	1.3	4.1	0.6	36.6
Total	74.8	2.2	69.5	12.9	2.5	10.3	2.6	100.0

CIHA, complementary and integrative health approaches. These data were excluded pregnant women and prescription or nonprescription medications for weight loss. Herbal medicines were included because they are classified as a complementary and alternative therapy in Korea. All variable values are measured and expressed in percentages. The ‘s’ is an abbreviation for school.

**Table 4:** CIHA for weight management according to general characteristics in Korean obese men population (≥19 years old).

Variables	Exercise	Fasting	Decreased Food Intake	Skipping Meals	Herbal Medicines	Health Functional Food	One-Food Diet	Total
Age group								
19–29	9.6	0.1	9.6	1.8	0.1	1.0	0.4	11.0
30–39	14.9	1.0	12.7	2.8	0.6	1.2	0.4	18.3
40–49	18.3	0.5	14.4	4.3	0.1	1.2	0.3	22.5
50–59	14.6	0.3	10.2	1.9	0.1	0.8	0.1	18.4
60–69	14.4	0.0	9.5	0.8	0.0	1.4	0.6	17.9
≥70	9.8	0.0	7.4	0.3	0.1	0.9	0.1	11.9
Total	81.6	1.9	63.7	11.9	1.2	6.5	1.9	100.0
Marital status								
Married	65.8	1.8	49.6	9.1	0.9	4.8	1.6	81.1
Single	15.8	0.1	14.1	2.8	0.3	1.7	0.4	18.9
Total	81.6	1.9	63.7	11.9	1.2	8.5	1.9	100.0
Economic activity status								
Yes	62.7	1.9	49.5	10.9	0.9	5.3	1.6	78.2
No	18.9	0.1	14.2	1.3	0.1	1.1	0.4	21.8
Total	81.5	2.0	63.7	12.2	1.1	6.4	2.0	100.0
Family income level								
Lower	7.8	0.3	5.9	1.3	0.1	0.7	0.0	10.5
Mid-lower	18.5	0.7	13.3	2.6	0.3	2.0	0.8	21.8
Mid-upper	25.9	0.4	20.0	3.0	0.5	1.8	0.7	32.2
Upper	29.4	0.7	24.8	5.1	0.3	2.0	0.5	35.4
Total	81.5	2.0	64.0	12.1	1.2	6.4	2.0	100.0
Education level								
≤Primary S.	7.0	0.0	4.9	0.7	0.0	0.5	0.3	8.9
Middle S.	7.0	0.0	4.9	0.1	0.0	0.3	0.1	8.8
High S.	28.4	0.5	22.3	4.0	0.4	3.3	1.2	36.0
College≤	39.0	1.5	31.6	7.4	0.7	2.3	0.4	46.3
Total	81.5	2.0	63.7	12.2	1.1	6.4	2.0	100.0

CIHA, complementary and integrative health approaches. Herbal medicines were included because they are classified as a complementary and alternative therapy in Korea. All variable values are measured and expressed in percentages. The ‘s’ is an abbreviation for school.

**Table 5:** CIHA for weight management according to general characteristics in Korean obese women population (≥19 years old).

Variables	Exercise	Fasting	Decreased Food Intake	Skipping Meals	Herbal Medicines	Health Functional Food	One-Food Diet	Total
Age group								
19–29	5.1	0.1	6.0	1.8	0.4	1.6	0.6	7.0
30–39	8.5	0.7	10.5	3.0	1.3	3.1	0.1	12.9
40–49	12.4	0.7	16.1	2.8	0.9	4.0	0.7	19.6
50–59	14.9	0.6	14.8	2.4	0.9	2.8	0.9	21.7
60–69	14.2	0.3	14.8	1.8	0.4	2.6	1.0	21.3
≥70	12.1	0.0	13.9	1.7	0.1	0.1	0.1	17.5
Total	67.2	2.4	76.0	13.6	4.0	14.2	3.4	100.0
Marital status								
Married	59.9	2.0	67.5	11.4	3.4	12.8	3.0	90.5
Single	7.2	0.4	8.5	2.3	0.6	1.4	0.4	9.5
Total	67.2	2.4	76.0	13.6	4.0	14.2	3.4	100.0
Economic activity status								
Yes	33.3	1.8	38.7	6.9	2.5	8.5	2.3	51.6
No	33.9	0.7	37.2	6.9	1.6	6.0	0.9	48.4
Total	67.2	2.5	76.0	13.8	4.1	14.5	3.2	100.0
Family income level								
Lower	16.0	0.4	18.1	3.7	0.1	1.6	0.6	24.0
Mid-lower	19.3	0.3	22.3	2.7	0.6	4.3	0.1	28.7
Mid-upper	18.3	1.1	21.5	4.4	1.6	4.9	0.9	27.0
Upper	13.8	0.6	14.1	2.9	1.7	3.6	1.0	20.4
Total	67.3	2.4	76.0	13.7	4.0	14.3	3.4	100.0
Education level								
≤Primary S.	14.7	0.1	18.9	2.8	0.6	0.9	0.6	24.8
Middle S.	10.0	0.1	18.9	2.8	0.6	0.9	0.6	14.4
High S.	24.5	0.9	26.8	5.1	1.3	5.9	1.3	34.8
College≤	18.0	1.3	20.8	4.5	1.9	6.2	0.7	26.1
Total	67.2	2.5	76.0	13.8	4.1	14.5	3.2	100.0

CIHA, complementary and integrative health approaches. This data excludes pregnant women and prescription or non-prescription medications for weight loss. Herbal medicines were included because they are classified as a complementary and alternative therapy in Korea. All variable values are measured and expressed in percentages. The “s” is an abbreviation for school.

## Data Availability

Restrictions apply to the availability of these data. Data was obtained from the Korea Disease Control and Prevention Agency (KDCA)] and are available https://knhanes.cdc.go.kr/knhanes/sub03/sub03_02_02.do (accessed on 30 March 2020) with the permission of the KDCA.
